# New Evidence on Breastfeeding and Postpartum Depression: The Importance of Understanding Women’s Intentions

**DOI:** 10.1007/s10995-014-1591-z

**Published:** 2014-08-21

**Authors:** Cristina Borra, Maria Iacovou, Almudena Sevilla

**Affiliations:** 1 Facultad de Ciencias Económicas y Empresariales, University of Seville, Ramón y Cajal 1, 41018 Seville, Spain; 2 Department of Sociology, University of Cambridge, Free School Lane, Cambridge, CB2 3RQ UK; 3 Queen Mary University of London, Mile End Road, London, E1 4NS UK; 4 ISER, University of Essex, Colchester, CO4 3SQ UK

**Keywords:** Breastfeeding, Mental health, Edinburgh postnatal depression scale, Child development, ALSPAC

## Abstract

This study aimed to identify the causal effect of breastfeeding on postpartum depression (PPD), using data on mothers from a British survey, the Avon Longitudinal Study of Parents and Children. Multivariate linear and logistic regressions were performed to investigate the effects of breastfeeding on mothers’ mental health measured at 8 weeks, 8, 21 and 32 months postpartum. The estimated effect of breastfeeding on PPD differed according to whether women had planned to breastfeed their babies, and by whether they had shown signs of depression during pregnancy. For mothers who were not depressed during pregnancy, the lowest risk of PPD was found among women who had planned to breastfeed, and who had actually breastfed their babies, while the highest risk was found among women who had planned to breastfeed and had not gone on to breastfeed. We conclude that the effect of breastfeeding on maternal depression is extremely heterogeneous, being mediated both by breastfeeding intentions during pregnancy and by mothers’ mental health during pregnancy. Our results underline the importance of providing expert breastfeeding support to women who want to breastfeed; but also, of providing compassionate support for women who had intended to breastfeed, but who find themselves unable to.

## Introduction

Approximately 13 % of women experience postpartum depression (PPD) within the 14 weeks after giving birth [1]. If the antenatal period is also considered, as many as 19 % of women experience a depressive episode during pregnancy or the first 3 months postpartum [2]. Post-natal depression has an immediate impact on mothers and carries long-term risks for mothers’ future mental health [3, 4]; it also has significant negative effects on the cognitive, social and physical development of their children [5, 6]. In addition, post-natal depression involves substantial economic costs, in terms of costs to healthcare systems [7] and losses in productivity via maternal absenteeism from work, premature retirement, and long-term unemployment [8].

The effect of breastfeeding on the risk of PPD is not well understood. Several studies have demonstrated an association between longer breastfeeding durations and a lower prevalence of PPD [9–14]. However, other studies have suggested the opposite, namely that breastfeeding mothers are at increased risk of PPD [15, 16]; or found no association [17, 18]. Of those studies which suggest beneficial effects from breastfeeding, several have relied on small samples, and few have controlled for potential confounders such as socioeconomic factors (maternal education, family income, marital status), the quality of relationships (marital stability, social networks), and stressful life events [19, 20]. Thus, it has been extremely difficult to identify whether the observed relationships are causal, as opposed to arising because breastfeeding is more likely to be practiced by mothers whose characteristics are themselves associated with a lower risk of depression [21–23]. Additionally, as Ip et al. [24] have pointed out, most existing studies have not controlled for pre-existing mental health conditions.

Thus, the extent to which breastfeeding influences mental health, as opposed to mental health driving the incidence and duration of breastfeeding, has not been clear. The aim of the current study is to examine explicitly whether breastfeeding affects maternal mental health outcomes. Specifically, we examine the hypothesis that the relationship between breastfeeding and maternal mental health is mediated by the mother’s intention to breastfeed. The relationship between breastfeeding and maternal mental health may be driven by biological factors, such as differences in hormone levels between breast- and formula-feeding mothers [25]; if maternal mental health is also affected by mothers’ feelings of success or failure in relation to their original plans and aspirations, we may expect the intention to breastfeed to play a crucial role.

## Data and Methods

### Data and Key Variables

This research is based on data from the Avon Longitudinal Survey of Parents and Children (ALSPAC), a survey of around 14,000 children born in the Bristol area of England in the early 1990s [26]. Mothers were recruited into the survey by doctors, at the point when they first reported their pregnancy. Data were collected by questionnaires administered to both parents at four points during pregnancy and at several stages following birth.

Details of all data collected in the ALSPAC survey are available on the study website through a fully searchable data dictionary [27]. Our study obtained ethical approval from the ALSPAC Law and Ethics Committee and the Local Research Ethics Committees.

We used a sample of mothers whose children form the “core sample” of ALSPAC. This sample consists of 14,541 pregnancies which resulted in 14,676 known foetuses; there were 14,062 live births, and 13,988 babies surviving to 1 year. We employed a maximizing strategy with respect to sample size, using as many observations as possible to analyse each outcome-effect dyad. Sample sizes thus vary slightly between regressions. The experiences of mothers and babies following pre-term births, or separation due to NICU care, may differ from the experiences of other mothers and babies. We do not exclude these mother/baby pairs from our sample, but have checked that our results do not change if they are excluded; these results are available from the authors on request.

As a measure of depression, the Edinburgh Postnatal Depression Scale (EPDS) was used. The EPDS, designed by Cox et al. [28] to screen for PPD, was collected during pregnancy at 18 and 32 weeks’ gestation, and post-natally at 8 weeks, and 8, 18, and 33 months. The EPDS is the most frequently used screening questionnaire for PPD; the EPDS is sensitive to changes in depression over time, and has been demonstrated to be a valid and reliable tool for the measurement of both postpartum and antenatal depression [29, 30]. The instrument consists of 10 questions, each with four possible answers describing symptoms of increasing severity or duration; aggregate scores on the EPDS range from 0 to 30. The authors of the EPDS have suggested that women should be referred to a mental health specialist if they score 13 or higher during the post-partum period [31] and 15 or more during pregnancy [32]. Therefore, we constructed indicators of depressive symptomatology, defined as EPDS >14 in pre-natal assessments and EPDS >12 in postpartum assessments.

Mothers were asked during pregnancy how they intended to feed their babies for the first 4 weeks. Following their child’s birth, they were asked at several points how they were actually feeding, and the ages at which infant formula and solid foods were introduced. Using this information, we computed seven binary indicators: (1) initiation (putting the baby to the breast at least once); (2–4) any breastfeeding for at least 1, 2 and 4 weeks respectively; and (5–7) exclusive breastfeeding for at least 1, 2 and 4 weeks respectively. We also computed two continuous indicators: total duration of breastfeeding and total duration of exclusive breastfeeding; results for these continuous indicators are similar to results obtained using the binary indicators, and are available from the authors on request.

### Analysis

We estimate multivariate logistic regressions, presenting odds ratios and 95 % confidence intervals. All hypotheses are tested using two-tailed *p* values <0.05.

We present estimates from three specifications. Model A controls only for the child’s sex and parental education. Model B additionally controls for other socio-demographic variables, and information on pregnancy and birth. Finally, Model C includes information on the mother’s physical and mental health, including antenatal EPDS assessments, together with factors relating to the quality of interpersonal relationships and stressful life events (see Table 6 in the Appendix for precise definitions of these variables). Thus, Model A provides a first approximation to the associations of interest, Model B estimates these relationships net of a range of potential confounders, while Model C aims to estimate causal relationships as accurately as possible by eliminating potential reverse causality arising from the fact that previously depression-prone mothers may be less likely to decide to breastfeed, or to breastfeed for shorter durations.

After conducting this analysis for the whole sample, we split the sample into mothers who were, and who were not, depressed during pregnancy; for each group, we examine differences in outcomes between women who had planned to breastfeed, and women who had not.

## Results

### Study Variables

Descriptive statistics for variables of interest are shown in Table 1. The prevalence of antenatal depression, using a cut-off of EPDS >14, is 7 % at 18 weeks’ pregnancy and 8 % at 32 weeks, similar to rates reported in previous studies [33]. Rates of PPD were between 9 and 12 %, also similar to results from former analyses [34].

**Table 1:** Descriptive statistics for variables of interest

	N	mean	s.d.
Maternal mental health during pregnancy			
At risk of antenatal depression, 18 weeks (EPDS >14)	10,904	7 %	(0.3)
At risk of antenatal depression, 32 weeks (EPDS >14)	11,305	8 %	(0.3)
Maternal mental health post-partum			
At risk of postpartum depression, 8 weeks (EPDS >12)	10,756	10 %	(0.3)
At risk of postpartum depression, 8 months (EPDS >12)	10,345	8 %	(0.3)
At risk of postpartum depression, 21 months (EPDS >12)	9,605	10 %	(0.3)
At risk of postpartum depression, 33 months (EPDS >12)	8,985	12 %	(0.3)
Breastfeeding			
Mother intended to breastfeed	11,547	65 %	(0.5)
Initiated breastfeeding	11,012	80 %	(0.4)
Breastfed for 1 week	10,668	74 %	(0.4)
Breastfed for 2 weeks	10,680	68 %	(0.5)
Breastfed for 4 weeks	10,972	56 %	(0.5)
Duration of any breastfeeding (months)	8,317	5.17	(4.7)
Exclusively breastfed for 1 week	10,668	64 %	(0.5)
Exclusively breastfed for 2 weeks	10,680	60 %	(0.5)
Exclusively breastfed for 4 weeks	10,972	43 %	(0.5)
Duration of exclusive breastfeeding (months)	8,726	1.31	(1.2)

Figures in the middle column are means in the case of continuous variables, and percentages of the sample in the case of dichotomous variables

80 % of mothers in this sample initiated breastfeeding and 74 % breastfed for 1 week or more. By 4 weeks only 56 % of mothers were breastfeeding at all and only 43 % were breastfeeding exclusively. The percentages of women feeding for the different durations considered are shown in Table 1; mean durations for breastfeeding and exclusive breastfeeding are also shown.

Table 2 shows the raw relationships between postnatal depressive symptomatology, and (a) prenatal depression, and (b) different measures of breastfeeding duration. A significant degree of correlation is present between postnatal and antenatal EPDS scores; a clear negative relationship also exists between symptoms of maternal depression measured at 8 weeks, and breastfeeding duration. The association between depression and breastfeeding is always negative, but generally statistically insignificant, at 8, 21 and 33 months.

**Table 2:** Raw correlations between study variables

	Postpartum EPDS scores			
	Postnatal EPDS >12	Postnatal EPDS >12	Postnatal EPDS >12	Postnatal EPDS >12
	at 8 weeks	at 8 months	at 21 months	at 33 months
Maternal mental health during pregnancy				
Antenatal EPDS >14 at 18 weeks	0.279***	0.220***	0.216***	0.207***
Antenatal EPDS >14 at 32 weeks	0.350***	0.309***	0.288***	0.271***
Breastfeeding measures				
Initiated breastfeeding	−0.034**	−0.027*	−0.018	−0.018
Breastfed for 1 week or more	−0.037**	−0.021	−0.019	−0.015
Breastfed for 2 weeks or more	−0.038**	−0.023	−0.015	−0.010
Breastfed for 4 weeks or more	−0.037**	−0.011	−0.005	−0.005
Duration of any breastfeeding	−0.044***	−0.021	−0.020	−0.009
Exclusively breastfed for 1 week or more	−0.041***	−0.019	−0.023	−0.022
Exclusively breastfed for 2 weeks or more	−0.040***	−0.033**	−0.018	−0.026*
Exclusively breastfed for 4 weeks or more	−0.052***	−0.021	−0.016	−0.013
Duration of exclusive breastfeeding	−0.036**	−0.025	−0.021	−0.014

*P* values are indicated by asterisks, with * *P* < 0.05, ** *P* < 0.01, *** *P* < 0.001

### Sample Characteristics

Socio-demographic characteristics for sample members are presented in Table 7 in the Appendix. The mean age of participants was 28.3 years (SD = 4.8). 95 % of the women were white, 86 % were married, 13 % had university degrees, while a further 22 % had high school qualifications at age 18 (“A” levels); and 74 % owned the house in which they lived. In relation to pregnancy and birth, 64 % felt usually well, 55 % percent were working while pregnant, 45 % were primiparous, and only 9 % delivered via Cesarean section. The average gestational age was 39.5 weeks (SD = 1.8). 48 % of mothers and 37 % of fathers had themselves been breastfed as babies. 28 % of the pregnancies were unplanned; 15 % of mothers had lived through their own parents’ divorce before their eighteenth birthday.

Table 3 presents the results of logistic regressions estimating the effect of breastfeeding on PPD.

**Table 3:** Results from logistic regressions: effects of breastfeeding on postpartum depression

	Model A Adjusted OR [95 % CI]	Model B Adjusted OR [95 % CI]	Model C Adjusted OR [95 % CI]
Dependent variable: EPDS >12 at 8 weeks			
Breastfeeding initiated	0.87 [0.74,1.03]	1.06 [0.88,1.27]	1.1 [0.89,1.37]
Any b/f, 1 week +	0.8 [0.69,0.93]**	0.95 [0.80,1.13]	1.08 [0.88,1.33]
Any b/f, 2 weeks +	0.83 [0.71,0.96]*	0.93 [0.78,1.09]	0.98 [0.81,1.19]
Any b/f, 4 weeks +	0.77 [0.67,0.89]***	0.81 [0.70,0.95]**	0.88 [0.74,1.06]
Exclusive b/f, 1 week +	0.8 [0.70,0.92]**	0.91 [0.78,1.06]	0.99 [0.82,1.19]
Exclusive b/f, 2 weeks +	0.78 [0.68,0.90]***	0.85 [0.73,0.99]*	0.89 [0.74,1.06]
Exclusive b/f, 4 weeks +	0.73 [0.64,0.85]***	0.75 [0.64,0.88]***	0.81 [0.68,0.97]*
N	10,509–10,546	10,393–10,428	9,722–9,757
Dependent variable: EPDS >12 at 8 months			
Breastfeeding initiated	0.86 [0.72,1.03]	1.01 [0.83,1.23]	0.99 [0.79,1.24]
Any b/f, 1 week +	0.9 [0.76,1.07]	1.04 [0.86,1.25]	1.15 [0.93,1.43]
Any b/f, 2 weeks +	0.88 [0.75,1.03]	0.98 [0.82,1.17]	1.02 [0.84,1.25]
Any b/f, 4 weeks +	0.89 [0.76,1.04]	0.95 [0.81,1.13]	1.05 [0.87,1.28]
Exclusive b/f, 1 week +	0.92 [0.79,1.07]	1.02 [0.86,1.21]	1.12 [0.92,1.36]
Exclusive b/f, 2 weeks +	0.83 [0.71,0.97]*	0.9 [0.76,1.06]	0.93 [0.77,1.12]
Exclusive b/f, 4 weeks +	0.86 [0.74,1.00]	0.9 [0.76,1.06]	1.02 [0.84,1.23]
N	10,080–10,116	9,258–9,999	9,354–9,388
Dependent variable: EPDS >12 at 21 months			
Breastfeeding initiated	0.93 [0.78,1.11]	1.08 [0.89,1.32]	1.09 [0.87,1.37]
Any b/f, 1 week +	0.97 [0.82,1.15]	1.14 [0.94,1.38]	1.26 [1.02,1.56]*
Any b/f, 2 weeks +	1 [0.86,1.18]	1.11 [0.93,1.33]	1.19 [0.97,1.46]
Any b/f, 4 weeks +	0.99 [0.85,1.14]	1.03 [0.87,1.21]	1.15 [0.95,1.38]
Exclusive b/f, 1 week +	0.93 [0.80,1.08]	1.04 [0.88,1.23]	1.19 [0.98,1.44]
Exclusive b/f, 2 weeks +	0.96 [0.82,1.11]	1.03 [0.87,1.21]	1.11 [0.92,1.33]
Exclusive b/f, 4 weeks +	0.9 [0.77,1.04]	0.92 [0.79,1.08]	1.06 [0.88,1.27]
N	9,370–9,406	9,258–9,929	8,704–8,737
Dependent variable: EPDS >12 at 33 months			
Breastfeeding initiated	1.04 [0.88,1.24]	1.22 [1.01,1.48]*	1.22 [0.98,1.51]
Any b/f, 1 week +	1.01 [0.86,1.18]	1.16 [0.97,1.39]	1.27 [1.04,1.55]*
Any b/f, 2 weeks +	1.02 [0.87,1.18]	1.13 [0.95,1.33]	1.19 [0.99,1.44]
Any b/f, 4 weeks +	1.01 [0.88,1.16]	1.07 [0.92,1.25]	1.17 [0.98,1.39]
Exclusive b/f, 1 week +	0.92 [0.80,1.06]	1.01 [0.86,1.18]	1.09 [0.92,1.30]
Exclusive b/f, 2 weeks +	0.9 [0.78,1.03]	0.96 [0.82,1.11]	0.99 [0.84,1.18]
Exclusive b/f, 4 weeks +	0.95 [0.83,1.09]	0.98 [0.85,1.14]	1.1 [0.93,1.29]
N	8,704–8,805	8,676–8,706	8,172–8,202

Coefficients are expressed as odds ratios and 95 % confidence intervals. Each estimated coefficient comes from a different regression. Model A controls for the child’s sex and parental education. Model B additionally controls for pregnancy and birth information; child characteristics at birth; demographic and socio-economic variables; and breastfeeding attitudes. Model C also controls for mother’s health in pregnancy, interpersonal relationships, and stressful life events (see Table 6 in the Appendix). Sample sizes vary slightly between regressions; the range of N is given in each panel

*P* values are indicated by asterisks, with * *P* < 0.05, ** *P* < 0.01, *** *P* < 0.001

As explained earlier, three models are estimated: Model A controls only for the child’s sex and parental education; Model B controls in addition for a wide range of socioeconomic and demographic factors, plus information on pregnancy and birth; and Model C also controls for mother’s health (including mental health) in pregnancy, relationship quality and stressful life events.

We consider four different outcomes: EPDS >12 measured at 8 weeks, 8, 21 and 33 months postpartum. For each model/outcome dyad, the model is estimated seven times, for seven different measures of breastfeeding (initiation; any breastfeeding for at least 1, 2 and 4 weeks; and exclusive breastfeeding for at least 1, 2 and 4 weeks). Thus, each coefficient in Table 3 comes from a separate regression.

At 8 weeks postpartum, we observe a pronounced relationship between breastfeeding and PPD, under both Models A and B. The odds ratios for these models indicate that longer durations of breastfeeding are associated with larger reductions in the risk of PPD, and exclusive breastfeeding is associated with a larger reduction than any breastfeeding. However, under Model C, when we control for mothers’ health during pregnancy, these effects largely disappear; the only significant relationship which remains comes from exclusive breastfeeding for 4 weeks or longer (OR 0.81, 95 % CI 0.68, 0.97).

The relationship between breastfeeding and PPD is also weaker, the later the EPDS score is assessed; at 8 months postpartum and thereafter, most of the estimated coefficients are not significantly different from zero (indeed, a few of the results are counter-intuitive, suggesting that breastfeeding may be *positively* related to an increased risk of depression measured at 33 months postpartum).

Thus, for the sample as a whole, our results demonstrate little evidence for a causal relationship between breastfeeding and the risk of PPD. In the next section, we investigate the possibility that the relationship between breastfeeding and depression varies according to two factors: whether mothers were assessed as at risk of depression during pregnancy, and whether they had been planning to breastfeed their babies. We show that the relationship between breastfeeding and depression is indeed highly heterogeneous, and that this fact explains why little effect is found when considering women as a homogeneous group.

### Heterogeneous Effects by Mental Health During Pregnancy and Breastfeeding Intention

We re-estimated Model C separately for mothers who were, and who were not, depressed during pregnancy (in terms of having a score EPDS >14 at least once during pregnancy). As before, we estimated regressions separately for each time at which postnatal depression was assessed (8 weeks, and 8, 21 and 33 months postpartum); for each of these time periods, we estimated seven models, one for each discrete measure of breastfeeding. However, instead of simply controlling for whether or not mothers breastfed for the relevant duration, we identify four groups of women, by whether they had *planned* to breastfeed, and whether they had *actually* breastfed for the relevant duration. These four groups are:

- Mothers who *had not* planned to breastfeed, and who *did not* breastfeed (reference group)
- Mothers who *had not* planned to breastfeed, but who *did* actually breastfeed
- Mothers who *had* planned to breastfeed, but who *did not* actually breastfeed
- Mothers who *had* planned to breastfeed, and who *did* actually breastfeed

Each regression thus generates three coefficients of interest; these coefficients are expressed as odds ratios, relative to the reference group.

Table 4 presents results for mothers *without* prenatal depression symptoms. Column (2) displays odds ratios and confidence intervals for mothers who did not plan to breastfeed, but who did actually breastfeed; column (3) indicates whether these mothers are significantly different from the mothers in the reference group.

**Table 4:** Results from logistic regressions: effects of breastfeeding on postpartum depression (mothers not at risk of depression during pregnancy)

Dependent variable		(1) Didn’t plan to breastfeed, didn’t breastfeed (reference group)	(2) Didn’t plan to breastfeed, did breastfeed		(3) Difference between coeffs (1) and (2)	(4) Planned to breastfeed, didn’t breastfeed		(5) Planned to breastfeed, did breastfeed		(6) Difference between coeffs (4) and (5)
EPDS > 12 at 8 weeks (N = 8,629–8,597)	Breastfeeding initiated	–	1.24	[0.88, 1.75]		2.55	[1.34,4.84]	0.36	[0.18, 0.71]	***
	Any b/f, 1 week +	–	1.33	[0.92, 1.92]		1.60	[0.97, 2.63]	0.54	[0.30, 0.96]	**
	Any b/f, 2 weeks +	–	1.43	[0.98, 2.09]		1.44	[0.96, 2.16]	0.56	[0.33, 0.94]	**
	Any b/f, 4 weeks +	–	1.26	[0.82, 1.94]		1.31	[0.96, 1.78]	0.61	[0.37, 1.01]	**
	Exclusive b/f, 1 week +	–	1.34	[0.91, 1.97]		1.45	[1.00, 2.09]	0.58	[0.35, 0.95]	**
	Exclusive b/f, 2 weeks +	–	1.30	[0.86, 1.96]		1.41	[1.01, 1.96]	0.58	[0.35, 0.96]	**
	Exclusive b/f, 4 weeks +	–	1.26	[0.74, 2.14]		1.22	[0.93, 1.59]	0.66	[0.37, 1.17]	*
EPDS > 12 at 8 months (N = 8,300–8,334)	Breastfeeding initiated	–	0.75	[0.51, 1.09]		1.37	[0.62, 3.03]	1.09	[0.46, 2.55]	
	Any b/f, 1 week +	–	0.89	[0.59, 1.35]		1.02	[0.57, 1.84]	1.37	[0.69, 2.72]	
	Any b/f, 2 weeks +	–	0.87	[0.56, 1.36]		1.47	[0.97, 2.24]	0.92	[0.52, 1.63]	
	Any b/f, 4 weeks +	–	0.80	[0.47, 1.34]		1.32	[0.95, 1.85]	1.13	[0.62, 2.03]	
	Exclusive b/f, 1 week +	–	1.00	[0.64, 1.55]		1.39	[0.94, 2.06]	0.95	[0.55, 1.66]	
	Exclusive b/f, 2 weeks +	–	0.77	[0.47, 1.26]		1.59	[1.13, 2.24]	0.93	[0.53, 1.66]	
	Exclusive b/f, 4 weeks +	–	0.66	[0.33, 1.31]		1.30	[0.97, 1.73]	1.49	[0.72, 3.08]	
EPDS > 12 at 21 months (N = 7,751–7,787)	Breastfeeding initiated	–	1.16	[0.82, 1.64]		1.67	[0.80, 3.48]	0.56	[0.26, 1.21]	
	Any b/f, 1 week +	–	1.44	[1.00, 2.08]	*	0.87	[0.49, 1.55]	0.95	[0.50, 1.81]	
	Any b/f, 2 weeks +	–	1.62	[1.12, 2.36]	*	1.18	[0.77, 1.80]	0.64	[0.38, 1.08]	
	Any b/f, 4 weeks +	–	1.61	[1.07, 2.43]	*	1.16	[0.84, 1.59]	0.61	[0.38, 1.00]	*
	Exclusive b/f, 1 week +	–	1.49	[1.02, 2.18]	*	1.00	[0.68, 1.48]	0.80	[0.48, 1.33]	
	Exclusive b/f, 2 weeks +	–	1.34	[0.90, 2.01]		1.08	[0.77, 1.52]	0.79	[0.48, 1.29]	
	Exclusive b/f, 4 weeks +	–	1.32	[0.79, 2.20]		1.07	[0.82, 1.40]	0.77	[0.44, 1.34]	
EPDS > 12 at 33 months (N = 7,300–7,330)	Breastfeeding initiated	–	1.12	[0.81, 1.55]		1.32	[0.63, 2.75]	0.92	[0.42, 1.98]	
	Any b/f, 1 week +	–	1.24	[0.89, 1.74]		1.27	[0.79, 2.06]	0.87	[0.50, 1.50]	
	Any b/f, 2 weeks +	–	1.45	[1.02, 2.05]	*	1.60	[1.11, 2.32]	0.62	[0.39, 0.99]	**
	Any b/f, 4 weeks +	–	1.23	[0.83, 1.82]		1.28	[0.96, 1.71]	0.87	[0.55, 1.38]	
	Exclusive b/f, 1 week +	–	1.31	[0.91, 1.87]		1.54	[1.11, 2.14]	0.66	[0.42, 1.04]	**
	Exclusive b/f, 2 weeks +	–	1.15	[0.78, 1.68]		1.59	[1.18, 2.13]	0.68	[0.43, 1.07]	**
	Exclusive b/f, 4 weeks +	–	1.05	[0.64, 1.73]		1.25	[0.97, 1.59]	1.02	[0.60, 1.75]	

Each row presents a set of three coefficients from the same regression; these are expressed as odds ratios relative to the reference group, with 95 % confidence intervals. The coefficients in each row come from a different regression. Model C is estimated, controlling for all variables in Table 6.Sample sizes vary slightly between regressions; the range of N is given in each panel

*P* values are indicated by asterisks, with * *P* < 0.05, ** *P* < 0.01, *** *P* < 0.001

Column (4) presents odds ratios for mothers who planned to breastfeed but who did not breastfeed for the relevant duration; Column (5) present odds ratios for mothers who planned to breastfeed, and who did breastfeed for the relevant duration. Column (6) indicates whether the odds ratios in Column (4) and (5) are significantly different from each other. Thus, the test results in Column (3) indicate whether breastfeeding makes a difference in the case of women who did not originally plan to breastfeed, while the tests in Column (6) indicate whether breastfeeding makes a difference in the case of mothers who had planned to breastfeed.

The strongest result from Table 4 is that breastfeeding is strongly associated with a lower risk of depression at 8 weeks postpartum, for women who had planned to breastfeed. The odds ratios in Column 4 are all well over 1, while the odds ratios in Column 5 are all well below 1; the differences between the two are statistically significant at the 1 % level or better for the first six measures of breastfeeding, and significant at the 5 % level for the remaining measure. The effects are smaller for later assessment periods. At 8 and 21 months, the odds ratios in Column 5 are lower than the odds ratios in Column 4 in almost all cases; however, the differences are not statistically significant. At 33 months, the differences are larger again, and are significant at the 1 % level for three of the seven measures of breastfeeding.

Interestingly, among the group of mothers who had not planned to breastfeed, the risk of depression was higher among women who went on to breastfeed. These differences are statistically significant for depression measured at 21 months, the largest being for any breastfeeding for 2 weeks on EPDS at 21 months (OR 1.62; 95 % CI 1.12, 2.36); at 8 weeks and 33 months the coefficients are all positive, though not generally significant at the 5 % level). To test whether our results were driven by a few mothers with very severe depressive symptoms, we repeated the analysis excluding those mothers with EPDS scores of 20 or more (the cut-off used in general practitioners’ guidelines [35] ); the results were virtually the same. We also investigated whether the effects depended on whether the mother was primiparous or multiparous, as suggested by [36]; again, the results were not affected.

Results for mothers who had been assessed as at risk of depression during pregnancy are shown in Table 5. For this group, results are less well defined, at least in part because of the smaller sample size. Our findings suggest that among women who had planned to breastfeed, breastfeeding is associated with a lower risk of PPD (as for mothers not depressed during pregnancy, although with a much smaller effect). However, for previously depressed mothers, there may also be a protective effect from breastfeeding when mothers had *not* planned to breastfeed. These results should be interpreted with caution: the only significant effect was found on EPDS measured at 8 weeks and for at least 4 weeks’ exclusive breastfeeding (OR 0.42; 95 % CI 0.20, 0.90).

**Table 5:** Results from logistic regressions: effects of breastfeeding on postpartum mental health (mothers at risk of depression when pregnant)

Dependent variable		(1) Didn’t plan to breastfeed, didn’t breastfeed (reference group)	(2) Didn’t plan to breastfeed, did acually breastfeed		(3) Difference between coeffs (1) and (2)	(4) Planned to breastfeed, didn’t breastfeed		(5) Planned to breastfeed, did breastfeed		(6) Difference between coeffs (4) and (5)
EPDS > 12 at 8 weeks (N = 1,124–1,128)	Breastfeeding initiated	–	1.24	[0.79,1.94]		0.82	[0.29,2.34]	1.11	[0.37,3.33]	
	Any b/f, 1 week +	–	1.33	[0.81,2.19]		1.45	[0.74,2.84]	0.56	[0.25,1.24]	
	Any b/f, 2 weeks +	–	1.06	[0.63,1.80]		1.71	[0.95,3.07]	0.47	[0.22,1.02]	**
	Any b/f, 4 weeks +	–	0.69	[0.37,1.28]		1.10	[0.73,1.67]	1.13	[0.54,2.35]	
	Exclusive b/f, 1 week +	–	1.14	[0.67,1.96]		1.29	[0.77,2.16]	0.67	[0.32,1.37]	
	Exclusive b/f, 2 weeks +	–	1.19	[0.68,2.09]		1.46	[0.91,2.35]	0.49	[0.24,0.99]	**
	Exclusive b/f, 4 weeks +	–	0.42	[0.20,0.90]	*	1.07	[0.74,1.53]	1.65	[0.71,3.83]	
EPDS > 12 at 8 months (N = 1,042–1,047)	Breastfeeding initiated	–	0.91	[0.56,1.45]		0.85	[0.28,2.55]	1.34	[0.42,4.28]	
	Any b/f, 1 week +	–	1.02	[0.60,1.72]		1.23	[0.59,2.54]	0.91	[0.38,2.14]	
	Any b/f, 2 weeks +	–	0.98	[0.56,1.71]		1.36	[0.72,2.57]	0.81	[0.36,1.84]	
	Any b/f, 4 weeks +	–	0.97	[0.52,1.83]		1.03	[0.66,1.61]	1.09	[0.51,2.32]	
	Exclusive b/f, 1 week +	–	1.00	[0.57,1.76]		1.04	[0.59,1.82]	1.12	[0.52,2.42]	
	Exclusive b/f, 2 weeks +	–	0.88	[0.49,1.60]		1.08	[0.65,1.80]	1.16	[0.54,2.47]	
	Exclusive b/f, 4 weeks +	–	0.86	[0.41,1.80]		1.06	[0.72,1.56]	1.10	[0.48,2.53]	
EPDS > 12 at 21 months (N = 941–945)	Breastfeeding initiated	–	1.02	[0.62,1.69]		0.32	[0.08,1.34]	3.12	[0.72,13.64]	
	Any b/f, 1 week +	–	1.07	[0.62,1.86]		0.76	[0.33,1.73]	1.26	[0.49,3.26]	
	Any b/f, 2 weeks +	–	1.11	[0.63,1.95]		1.00	[0.49,2.01]	0.91	[0.38,2.19]	
	Any b/f, 4 weeks +	–	0.84	[0.44,1.60]		0.75	[0.47,1.22]	1.68	[0.76,3.70]	
	Exclusive b/f, 1 week +	–	0.89	[0.49,1.61]		0.74	[0.40,1.36]	1.50	[0.66,3.42]	
	Exclusive b/f, 2 weeks +	–	1.03	[0.56,1.89]		0.95	[0.55,1.65]	1.01	[0.46,2.24]	
	Exclusive b/f, 4 weeks +	–	0.64	[0.30,1.37]		0.82	[0.54,1.23]	1.93	[0.82,4.56]	
EPDS > 12 at 33 months (N–865–869)	Breastfeeding initiated	–	1.28	[0.76,2.15]		1.81	[0.53,6.16]	0.61	[0.17,2.18]	
	Any b/f, 1 week +	–	1.09	[0.62,1.92]		1.16	[0.53,2.55]	1.03	[0.41,2.59]	
	Any b/f, 2 weeks +	–	1.16	[0.65,2.08]		1.70	[0.88,3.30]	0.62	[0.27,1.45]	
	Any b/f, 4 weeks +	–	1.08	[0.56,2.08]		1.29	[0.80,2.08]	0.90	[0.41,1.96]	
	Exclusive b/f, 1 week +	–	1.01	[0.55,1.84]		1.33	[0.75,2.37]	0.92	[0.42,2.03]	
	Exclusive b/f, 2 weeks +	–	1.18	[0.64,2.20]		1.56	[0.92,2.65]	0.65	[0.30,1.41]	
	Exclusive b/f, 4 weeks +	–	0.97	[0.45,2.09]		1.28	[0.84,1.95]	0.97	[0.41,2.30]	

Each row presents a set of three coefficients from the same regression; these are expressed as odds ratios relative to the reference group, with 95 % confidence intervals. The coefficients in each row come from a different regression. Model C is estimated, controlling for all variables in Table 6.Sample sizes vary slightly between regressions; the range of N is given in each panel

*P* values are indicated by asterisks, with * *P* < 0.05, ** *P* < 0.01, *** *P* < 0.001

## Discussion

The aim of this study was to examine whether breastfeeding influenced the risks of postnatal depression. This study extends previous research by using a large longitudinal dataset; controlling for a large set of socioeconomic, relational, and psychosocial confounders; measuring maternal mood at different time points both before and after delivery; and utilising several measures of breastfeeding initiation, duration, and exclusivity.

We found that the effect of breastfeeding on maternal mood differed by both maternal mental health during pregnancy; and whether mothers intended to breastfeed. To our knowledge, this study is the first to document this result.

For the majority of mothers who did not show symptoms of depression before birth, breastfeeding decreased the risk of PPD among mothers who had intended to breastfeed, but increased the risk of PPD among mothers who had *not* intended to breastfeed.

We also found that the beneficial effects of breastfeeding were strongest at 8 weeks after birth, and that the association was weaker at 8 months and onwards. This finding is in line with the findings of the only other longitudinal research in this area [37] which significant effects at 6 weeks but not at 12 weeks postpartum. Our results are nevertheless important, because of the established relationship between depression, even in the very early postpartum period, and maternal-infant bonding [38].

Estimates for the smaller group of mothers who had shown signs of depression during pregnancy were less precise, but differed from the estimates for non-depressed women in two important ways. The protective effects of breastfeeding as planned were smaller for women who had been depressed during pregnancy; but exclusive breastfeeding for 4 weeks appeared to exercise a protective effect for this group, which it did not do for the women who had not been depressed in pregnancy.

We recognize several limitations in our analyses. Although we employ the most commonly used measure of depressive symptomatology, we acknowledge that including a clinical diagnosis of antenatal and PPD would have increased the value of our findings. Also, misclassification bias may arise when relying on self-report methods to assess breastfeeding outcomes. Thirdly, even though we use a large population-based sample with low loss to follow-up, sampling bias resulting from the voluntary nature of participation in the survey could have influenced results. For instance, we acknowledge a shortfall in the numbers of ethnic minority mothers that may limit the generalizability of the results. Finally, even though we control for many more potential confounders than any other study on the subject, there may remain some unobserved factor, for example aspects of maternal IQ or personality, which could affect the results.

In summary, the effect of breastfeeding on maternal depression symptoms was found to be highly heterogeneous and, crucially, mediated by breastfeeding intentions during pregnancy. Our most important finding relates to the majority of mothers who were not depressed during pregnancy, and who planned to breastfeed their babies. For these mothers, breastfeeding as planned decreased the risks of PPD, while not being able to breastfeed as planned increased the risks. These findings have implications for the way in which new mothers are supported; they suggest that the provision of expert breastfeeding support may, in addition to increasing breastfeeding rates and durations, have the additional benefit of improving mental health outcomes among new mothers. At the same time, it is clear that where mothers had intended to breastfeed, not being able to breastfeed may have deleterious consequences on their risk of PPD, and that providing specialised support to new mothers who had intended to breastfeed, but who for some reason find themselves unable to breastfeed, may also constitute a desirable health policy objective.

## Appendix 1

See Table 6.

**Table 6:** List of variables used in the analysis

Socio-demographic variables (at or during pregnancy)
Two dummies for housing tenure which take the value 1 if the mother owned the house or rented the house during pregnancy (omitted category is social housing); the number of rooms in the house during pregnancy; neighborhood indicators with higher values indicating a better neighbourhood; a dummy indicating the mother’s race (white, with omitted category nonwhite); three dummies indicating the marital status of the mother at the time of pregnancy (married, cohabiting, single/separated/divorced); five dummies indicating the mother’s and father’s education level (university degree; A levels (school qualifications obtained at age 18); O levels (school qualifications obtained at age 16); CSE (a lower level of school qualifications obtained at age 16) and vocational); and an indicator variable that takes the value 1 if the mother was working at 18 weeks of pregnancy.
Pregnancy and delivery information
A dummy that takes value 1 if the child is a female; a dummy that takes value 1 if the child is a twin; mother’s age at birth; an indicator variable that takes value 1 if the mother had a cesarean section; the length of the gestation period.
Health variables
Dummy variables for different physical health levels; number of cigarettes smoked each day measured at 32 weeks of pregnancy; number of alcoholic beverages a day before pregnancy; and antenatal EPDS measured at 18 and 33 months pregnancy.
Interpersonal relationships, personality, and stressful life events
Dragona’s et al. (1992) measure of the mother’s social network availability; Quinton and Rutter’s (1988) aggression and affection scores for marital quality; a psychological measure of the mother’s personality: the adult version of the Nowicki-Strickand locus of control scale (Duke and Nowicki, 1973); Barnett et al.’s (1983) Life Events Score; an indicator variable that takes the value 1 if pregnancy was unplanned; an indicator variable that takes value 1 if the mother was in local authority care; an indicator variable that takes value 1 if she had divorced parents by age 17; an indicator variable that takes value 1 if the mother’s main carer died by age 17;

## Appendix 2

See Table 7.

**Table 7:** Socio-demographic characteristics of study population

	Units	Mean	(Std. error)
Pregnancy and birth			
Gestation in weeks	Weeks	39.47	(1.8)
Mother’s age at birth	Years	28.34	(4.8)
C-section	0/1	0.09	(0.3)
Primiparous	0/1	0.45	(0.5)
Mother works at 18 weeks	0/1	0.55	(0.5)
Cigarettes at 32 w	No.	2.00	(5.1)
Previous alcohol consumption	No.	2.59	(0.8)
Child characteristics at birth			
Female	0/1	0.49	(0.5)
Twin	0/1	0.01	(0.1)
Birth weight	grams	3,419.93	(543.9)
Head circumference	inches	34.84	(1.4)
Crown-heel length	inches	50.52	(2.2)
Demographic and socio-economic variables			
White mother	0/1	0.95	(0.2)
Mother cohabiting	0/1	0.20	(0.4)
Mother single	0/1	0.04	(0.2)
Owner occupier	0/1	0.74	(0.4)
Private rented	0/1	0.07	(0.3)
Number of rooms	0/1	1.59	(0.9)
Neighbourhood qual.	0/1	8.25	(2.2)
Father’s education			
University degree	0/1	0.17	(0.4)
A level (academic qualifications age 18)	0/1	0.25	(0.4)
O level (academic qualifications age 16)	0/1	0.35	(0.5)
CSE (lower-level academic quals)	0/1	0.15	(0.4)
Vocational	0/1	0.08	(0.3)
Mother’s education			
University degree	0/1	0.13	(0.3)
A level (academic qualifications age 18)	0/1	0.22	(0.4)
O level (academic qualifications age 16)	0/1	0.42	(0.5)
CSE (lower-level academic quals)	0/1	0.14	(0.3)
Vocational	0/1	0.10	(0.3)
Breastfeeding attitudes			
Mother was breastfed	0/1	0.48	(0.5)
Father was breastfed	0/1	0.32	(0.5)
Father breastfeeding attitudes	score	15.39	(2.4)
Mother’s health in pregnancy			
Mother health always well	0/1	0.29	(0.5)
Mother health usually well	0/1	0.64	(0.5)
Mother health sometimes unwell	0/1	0.06	(0.2)
Mother health often unwell	0/1	0.01	(0.1)
Interpersonal relationships and stressful life events			
Mother’s social network score	score	23.34	(3.8)
Mother’s affection score	score	11.17	(4.2)
Mother’s aggression score	score	10.00	(1.7)
Mother’s std. locus/control score	score	0.00	(1.1)
Mother’s life events score	score	8.05	(7.3)
This pregnancy unplanned	0/1	0.28	(0.4)
Mother in care	0/1	0.02	(0.2)
Mother’s parents divorced by 17	0/1	0.15	(0.4)
Mother’s parents died by 17	0/1	0.10	(0.3)
